# Prognostic significance of leukopenia in childhood acute lymphoblastic leukemia

**DOI:** 10.3892/ol.2014.1822

**Published:** 2014-01-24

**Authors:** YUSUKE SHIOZAWA, JUNKO TAKITA, MOTOHIRO KATO, MANABU SOTOMATSU, KATSUYOSHI KOH, KOHMEI IDA, YASUHIDE HAYASHI

**Affiliations:** 1 Department of Pediatrics, The University of Tokyo Hospital, Tokyo 113-8655, Japan; 2 Department of Cell Therapy and Transplantation Medicine, The University of Tokyo Hospital, Tokyo 113-8655, Japan; 3 Department of Hematology/Oncology, Saitama Children’s Medical Center, Saitama 339-0077, Japan; 4 Department of Hematology-Oncology, Gunma Children’s Medical Center, Shibukawa, Gunma 377-0061, Japan; 5 Department of Pediatrics, Teikyo University Mizonokuchi Hospital, Kawasaki, Kanagawa 213-8507, Japan

**Keywords:** childhood acute lymphoblastic leukemia, chemotherapy, white blood cell count

## Abstract

Chemotherapy-induced leukopenia has been shown to be associated with the outcomes of several types of cancer, but the association with childhood acute lymphoblastic leukemia (ALL) remains unknown. To elucidate the association of chemotherapy-induced leukopenia with the clinical outcome of childhood ALL, retrospective analysis was performed on 19 child patients with ALL treated according to the ALL-BFM 95 high-risk (HR) protocol. The mean minimum leukocyte count over the first three courses of the consolidation phase was used as the measure of hematological toxicity and ranged between 200 and 1,167/μl. The risk of relapse was significantly higher in patients with a mean minimum leukocyte count above the median of 433/μl (hazard ratio, 6.61; P=0.047). In conclusion, chemotherapy-induced leukopenia was found to correlate with relapse-free survival in childhood HR ALL. Dose escalation based on hematologic toxicity must be prospectively studied.

## Introduction

Treatment outcome of childhood acute lymphoblastic leukemia (ALL) has evidently improved, but the prognosis of high-risk (HR) ALL remains unsatisfactory (1). Refinement of risk stratification is required to improve survival by providing intensive treatment to patients at HR of relapse. The clinical outcome of ALL is known to be associated with variable factors, such as demographics, immunophenotype, cytogenetic features and early treatment response (2). In addition, the ability of individual patients to metabolize antileukemic drugs appears to be involved in the prognosis of ALL, but the knowledge of pharmacological features of leukemic cells in childhood ALL is largely limited (3).

Hematological toxicity is the most frequent dose-limiting side effect of combination chemotherapy in the treatment of childhood ALL. The severity of each case of acute hematological toxicity is highly variable despite use of the same regimen. Chemotherapy-induced leukopenia may be a biological measure of drug activities and disease control (4,5). The response of leukemic cells to chemotherapy depends on the level of active drugs reaching the target and the sensitivity to these drugs. These factors also affect the response of non-malignant hematopoietic cells. The availability of active drugs is influenced by pharmacokinetic parameters. In part, sensitivity to the drugs is affected by genetic predisposition, which produces a similar effect in tumor and normal cells, but is also modified by tumor-specific mutations (6).

The association between less chemotherapy-induced leukopenia and poor clinical outcome has been previously reported for several malignancies, including lung cancer, breast cancer, osteosarcoma and Hodgkin lymphoma (6–12). This provides additional prognostic information that may be used to further refine patient stratification and risk-directed therapy. However, the prognostic role of chemotherapy-induced leukopenia in childhood ALL has not been elucidated.

Conventional treatment for ALL consists of induction, consolidation, reinduction and maintenance elements. Cytotoxic agents, dose levels and severity of myelosuppression are significantly different between treatment courses. This makes it difficult to define the measure of hematological toxicity compared with the treatment and to evaluate the prognostic significance of chemotherapy-induced leukopenia. In the ALL-BFM 95 HR protocol for childhood ALL, the consolidation phase consists of a series of intensive treatment courses (block therapy), with an interval of three to four weeks between blocks (13). This repetition of treatments with relatively similar intensity is suitable for the evaluation of chemotherapy-induced leukopenia. Therefore, to investigate the association of leukopenia early in the course of treatment with treatment outcomes of childhood ALL, the current study analyzed ALL patients treated according to the ALL-BFM 95 HR protocol following induction therapy.

## Materials and methods

### Study population

In total, 19 patients (age range, 1–18 years) consecutively diagnosed with ALL between November 2003 and September 2010 were studied and uniformly treated according to the ALL-BFM 95 HR protocol following induction therapy at the University of Tokyo Hospital (Tokyo, Japan), Saitama Children’s Medical Center (Saitama, Japan) and Gunma Children’s Medical Center (Shibukawa, Japan).

Children diagnosed with ALL were enrolled in the Tokyo Children’s Cancer Study Group (TCCSG) L99-1502 study between November 2003 and January 2005, and in the L04-16 study between May 2005 and September 2010 (14). The patients were stratified into the following three risk groups: Standard-risk (SR), intermediate-risk (IR) and HR. The initial stratification was based on presenting features (age and white blood cell count prior to initiating treatment) and leukemic blasts in peripheral blood on day eight following prednisolone monotherapy (Tables I and II). The patients were finally stratified based on cytogenetic observations and bone marrow status examined following remission induction therapy. Following induction therapy, patients assigned to the HR group were treated with block chemotherapy regimen of the ALL-BFM 95 HR protocol. Patients who did not achieve remission and those with the Philadelphia chromosome or 11q23 rearrangements (with the exception of MLL/ENL in the L04-16 study) were scheduled for allogeneic stem cell transplantation and were excluded from the present study.

The data regarding chemotherapeutic dosage, dates of administration and leukocyte counts were retrieved from the electronic patient databases of the hospitals involved. The parents of all patients provided written informed consent for the treatment. The current study was approved by the Ethics Committee of the University of Tokyo Hospital.

### Treatment protocols

**Figure 1:**
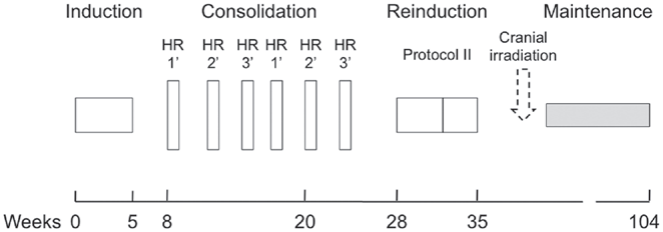
Outline of the treatment regimens. The treatment regimens consisted of induction, consolidation, reinduction and maintenance elements. The mean minimum leukocyte count over the first three courses of the consolidation phase was used as the measure of hematological toxicity. Cranial irradiation was administered only to patients with an initial leukocyte count of >100×10^9^/l in the L99-1502 study. Patients aged 1–6 years received 12 Gy and patients >6 years received 18 Gy. The indication of cranial irradiation was limited to patients with central nervous system involvement (12 Gy for patients aged 12–23 months and 18 Gy for patients aged ≥24 months) and T-lineage acute lymphoblastic leukemia patients with <1,000 leukemic blasts/μl on day 8 (12 Gy) in the L04-16 study. Remaining patients received no cranial irradiation. HR, high-risk.

An outline of the treatment regimens is shown in Fig. 1 and the details of each treatment aspect are provided in Table III (13). Following TCCSG induction therapy, patients were uniformly treated according to the ALL-BFM 95 HR protocol; the patients continued on an intensive rotational consolidation schedule consisting of three separate six-day pulses of high-dose chemotherapy, which were each administered twice. Patients were treated according to the reinduction protocol II following the consolidation phase.

Granulocyte colony-stimulating factor (G-CSF) was administered in certain patients with febrile neutropenia and occasionally used prophylactically when severe and prolonged neutropenia was predicted.

### Leukocyte count

Blood examination was routinely performed several times a week. The minimum leukocyte count during each course of chemotherapy was recorded. The minimum leukocyte count was averaged over the first three courses of the consolidation phase. The mean was used as the measure of hematological toxicity for each patient. The leukocyte count during the induction phase was excluded from the analysis, since disease status markedly affected the leukocyte count until remission was achieved.

### Study outcomes

To assess the correlation between leukocyte nadir and disease control, relapse-free survival (RFS) from the initiation of chemotherapy was selected as the endpoint.

### Statistical analysis

RFS curves were calculated by the Kaplan-Meier method and were compared by means of the log-rank test in a univariate analysis. The minimum leukocyte count in treatment courses with or without the use of G-CSF was compared with the Mann-Whitney U test to assess the effect of G-CSF on leukocyte nadir.

All statistical tests were two-tailed and P<0.05 was considered to indicate a statistically significant difference. Statistical analyses were performed using R software (R Foundation for Statistical Computing*,* Vienna, Austria).

## Results

### Patient characteristics

In total, 22 patients were assigned to the HR group on day eight in remission induction therapy. One patient in the SR group on day eight was stratified into the HR group due to residual leukemic infiltration in the liver and frontal bone, although, hematological remission was achieved. Finally, 23 patients were stratified into the HR group. Of these, four received allogeneic stem cell transplantation and were excluded from the analysis; one with the Philadelphia chromosome and three with a poor response to prednisolone. The remaining 19 patients were uniformly treated according to the ALL-BFM 95 HR protocol and included in the analysis. The median age was 11 years (range, 1–18 years) and eight (42%) patients were female. All patients were treated with the same dose per body surface area in the consolidation phase with the exception of L-asparaginase, which was not administered to two patients in the third course due to anaphylaxis. Detailed patient characteristics are shown in Table IV.

### Treatment outcome

Of the 19 patients, five suffered a relapse: Two during consolidation, one during reinduction and two during maintenance therapy. The median follow-up period of relapse-free patients was 51.5 months (range, 10–85 months). The mean minimum leukocyte count was calculated for the first three courses of the consolidation phase, with the exception of two patients who relapsed during the third course of the consolidation phase; their mean minimum leukocyte count was calculated for the first two courses of the consolidation phase. The median of the mean minimum leukocyte count was 433/μl (range, 200–1,167/μl).

Of the 19 patients, 13 received G-CSF at least once during treatment. The minimum leukocyte count was not significantly different between the courses with and without the use of G-CSF (P=0.367; Mann-Whitney U test).

### Prognostic factors

**Figure 2:**
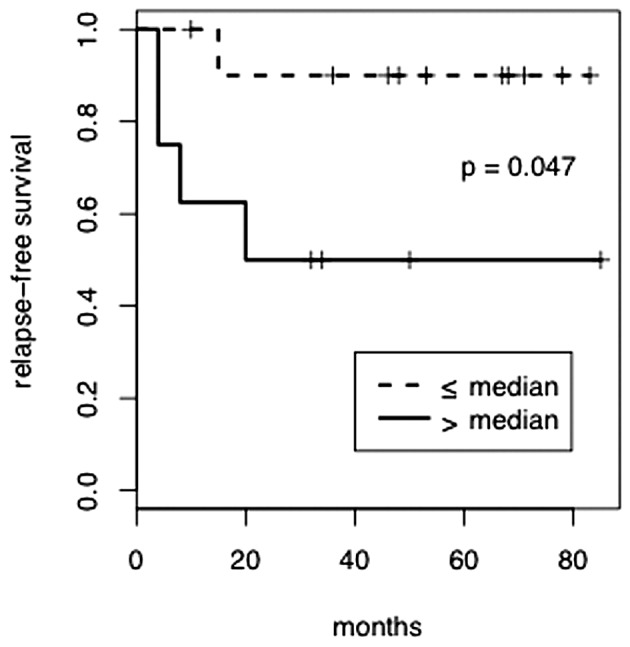
RFS according to the mean minimum leukocyte count. RFS is shown in patients with the mean minimum leukocyte count above the median of 433/μl (solid line) and those at or below the median (dashed line). RFS, relapse-free survival.

RFS curves were compared by means of the log-rank test in a univariate analysis (Table V). Variables included age, gender, immunophenotypes of leukemic blasts (B- or T-lineage), initial leukocyte count, response to prednisolone monotherapy and the mean minimum leukocyte count. Patients were divided at the median values for age, initial leukocyte count and the mean minimum leukocyte count. The risk of relapse was significantly higher in patients with a mean minimum leukocyte count above the median (hazard ratio, 6.61; P=0.047). Fig. 2 shows RFS according to the severity of leukopenia. No other factors were significantly associated with the risk of relapse.

## Discussion

The severity of acute hematological toxicity varies considerably in childhood ALL despite the use of the same chemotherapy. The current study analyzed patients with ALL in the same risk group and showed that patients with low hematological toxicity during chemotherapy exhibited a higher rate of relapse. HR of relapse was identified by low hematotoxicity in the first half of the consolidation phase. Early identification of the HR population enables us to intensify treatment in these patients.

Low hematological toxicity has been reported to be associated with a poorer outcome of other malignancies (6–12). This association is predicted to be evident in acute leukemia, considering the common origin of leukemic blasts and normal hematopoietic cells. Previously, Han *et al* showed that a leukocyte nadir of >1,200/μl in induction chemotherapy is associated with poor overall survival in adult patients with acute myeloid leukemia (AML), although, no statistically significant difference was identified (15). This is consistent with the observations of the current study. On the other hand, previous studies have reported that patients with severe hematological toxicity and a slow rate of myeloid recovery in induction chemotherapy exhibit a poor clinical outcome in adult AML and childhood ALL (15,16). The mechanism underlying this association is unclear, but leukemic blasts in bone marrow are likely to affect the leukocyte count until remission and rate of myeloid recovery following induction therapy. In the present study, chemosensitivity of non-malignant hematopoietic cells were evaluated following remission induction, when the effect of residual leukemic cells may almost be ignored.

A false association between leukopenia and treatment outcome may have been established, since more severe leukopenia was predicted, as the patients had prolonged survival and received more treatment courses. In the present cohort, 17 of the 19 patients completed all three courses of the first half of the consolidation phase. The remaining two patients who relapsed in the third course also received two out of three courses. Low hematological toxicity could not be fully explained by a reduced number of chemotherapy courses.

The results of the present study indicated that leukopenia may be used as a biomarker for effective chemotherapy dose, supporting the theory of individualizing chemotherapy dosage based on hematological toxicity (17). Patients with low acute hematological toxicity may be rapid metabolizers of cytotoxic agents. Considering that the hematopoietic cells of these patients exhibit low sensitivity to cytotoxic agents, corresponding leukemic blasts may also demonstrate low sensitivity to the drugs. Whether the outcome of these patients may be improved by dose-escalation must be prospectively studied in a large clinical trial.

The current study was unable to evaluate the influence of other possible prognostic factors by multivariate analysis, as the number of patients was too small. However, patients in the present cohort were stratified into the same risk group and were roughly adjusted for the conventional factors, including age, leukocyte count at diagnosis, immunophenotypes of leukemic blasts and early treatment response. This may be one of the reasons why these factors were not associated with relapse. In addition, chemotherapy-induced leukopenia is unlike the conventional risk factors, since it reflects the response of normal hematopoietic cells, but not tumor cells. Leukocyte nadir is thus predicted to be an independent prognostic factor. Further investigation in a larger cohort is required to assess this possibility.

In conclusion, the degree of chemotherapy-induced leukopenia was found to correlate with RFS in child patients with ALL. Trials exploring intrapatient dose escalation are warranted.

## Figures and Tables

**Table I:** Risk stratification (the TCCSG L99-1502 study).

A, B-lineage ALL			

	Years		
	
Initial risk	1–6	7–9	≥10
Initial leukocyte count, ×10^9^/l			
<20	SR	IR	IR
20–49	IR	IR	IR
50–99	IR	IR	HR
≥100	HR	HR	HR

B, B-lineage ALL			

	Days		
	
Day 8 risk	1 SR	1 IR	1 HR

Day 8 PB blasts/μl			
0	SR	IR	IR
1–999	SR	IR	HR
≥1,000	IR	HR	Allo-SCT

C, T-lineage ALL			

Day 8 risk			All patients

Day 8 PB blasts/μl			
0			IR
1–999			HR
≥1,000			Allo-SCT

PB, peripheral blood; SR, standard-risk; IR, intermediate-risk; HR, high-risk; Allo-SCT, allogeneic stem cell transplantation; ALL; acute lymphoblastic leukemia.

**Table II:** Risk stratification (the TCCSG L04-16 Study).

A, B-lineage ALL			

	Years		
	
Initial risk	1–6	7–9	≥10
Initial leukocyte count, ×10^9^/l			
<20	SR	IR	IR
20–49	IR	IR	IR
50–99	IR	IR	HR
≥100	HR	HR	HR

B, B-lineage ALL			

	Days		
	
Day 8 risk	1 SR	1 IR	1 HR

Day 8 PB blasts/μl			
0–999	SR^a^	IR^a^	HR
≥1,000	HR	HR	Allo-SCT

C, T-lineage ALL			

Day 8 risk			All patients

Day 8 PB blasts/μl			
0–999			HR
≥1,000			Allo-SCT

a Patients were assigned to high-risk group if >5 cells/μl were counted in the cerebrospinal fluid and lymphoblasts were identified or if intracranial infiltrates were detected on brain imaging studies.

Diagnostic lumbar puncture was performed on day eight in remission induction therapy. PB, peripheral blood; SR, standard-risk; IR, intermediate-risk; HR, high-risk; Allo-SCT, allogeneic stem cell transplantation.

**Table III:** Details of the treatment regimens.

Therapy	Details
Induction	
Day 8 SR	Pred, 60 mg/m^2^ × 5 weeks; VCR, 1.5 mg/m^2^ on weeks 1–5; Pirarubicin, 20 mg/m^2^ on weeks 3 and 4; and L-asp, 6,000 U/m^2^ 3 times a week on weeks 2–4
Day 8 IR and HR	Pred, 60 mg/m^2^ × 5 weeks; VCR, 1.5 mg/m^2^ on weeks 1–5; DNR, 25 mg/m^2^ 2 times a week on weeks 2 and 5; CY 1,000 mg/m^2^ on weeks 2 and 5; and L-asp 6,000 U/m^2^ 3 times a week on weeks 2–4
HR-1′ (two cycles)	Dex, 20 mg/m^2^ × 5 days; MTX, 5 g/m^2^ on day 1; CY, 200 mg/m^2^ once on day 2 and twice on days 3 and 4; Ara-C, 2 g/m^2^ twice on day 5; and L-asp, 25,000 U/m^2^ on day 6 (VCR 1.5 mg/m^2^ on day 1 and 6 only in the second cycle)
HR-2′ (two cycles)	Dex, 20 mg/m^2^ × 5 days; Vindesine, 3 mg/m^2^ on days 1 and 6; MTX, 5 g/m^2^; IFO, 800 mg/m^2^ once on day 2 and twice on days 3 and 4; DNR, 30 mg/m^2^ on day 5; and L-asp, 25,000 U/m^2^ on day 6
HR-3′ (two cycles)	Dex, 20 mg/m^2^ × 5 days; Ara-C, 2 g/m^2^ twice on days 1 and 2; VP-16, 100 mg/m^2^ once on day 3 and twice on days 4 and 5; and L-asp 25,000 U/m^2^ on day 6
Protocol II	
First half	Dex, 10 mg/m^2^ × 14 days; VCR, 1.5 mg/m^2^ on days 8, 15, 22 and 29; ADR, 30 mg/m^2^ on days 8, 15, 22 and 29; and L-asp, 10,000 U/m^2^ on days 3, 8, 16 and 21
Second half	6MP, 60 mg/m^2^ × 14 days; CY, 1 g/m^2^ on day 36; and Ara-C, 75 mg/m^2^ × 4 consecutive days for 2 weeks
Cranial irradiation	
Maintenance	6MP/MTX until week 104
Total number of IT therapies	10–17

SR, standard-risk; IR, intermediate-risk; HR, high-risk; Pred, prednisolone; VCR, vincristine; L-Asp, L-asparaginase; DNR, daunorubicin; CY, cyclophosphamide; Dex, dexamethasone; MTX, methotrexate; Ara-C, cytarabine; IFO, ifosfamide; VP-16, etoposide; ADR, adriamycin; 6MP, 6-mercaptopurine; IT, intrathecal.

**Table IV:** Characteristics of the study population.

No.	Age, years	Gender	Immunophenotype	Initial WBC, ×10^9^/l	Day 8 PB blast/μl	Risk group		Mean minimum WBC/μl	Outcome

						Day 1	Day 8		
1	12	M	B	81	63	HR	HR	1,167	RFS
2	11	F	B	581	632	HR	HR	450	Relapsed
3	14	M	T	279	14	HR	HR	433	RFS
4	8	F	B	7.2	7,684	IR	HR	367	RFS
5	9	M	T	430	116	HR	HR	500	Relapsed
6	12	F	B	8.2	1,269	IR	HR	733	Relapsed
7	14	M	T	1.5	0	HR	HR	367	RFS
8	7	M	T	259	20	HR	HR	433	Relapsed
9	12	F	T	147	247	HR	HR	233	RFS
10	15	M	T	42	0	HR	HR	433	RFS
11	6	F	T	11	0	HR	HR	233	RFS
12^a^	3	F	B	8.5	825	SR	SR	667	RFS
13	7	F	B	12	76	HR	HR	300	RFS
14	11	F	B	21	12,802	IR	HR	200	RFS
15	13	M	T	126	bND	HR	HR	633	Relapsed
16	10	M	B	539	7	HR	HR	467	RFS
17	13	M	B	53	81	HR	HR	200	RFS
18	6	M	T	28	28	HR	HR	467	RFS
19	6	M	T	65	459	HR	HR	300	RFS
Median	11			52.9	69.5			433	
IQR	6–13			8.5–278.6	7–825			233–633	

a Patient number 12 started treatment with the TCCSG L04-16 SR protocol. Hematological remission was achieved following remission induction, but leukemic infiltration remained in the liver and frontal bone. The patient was finally stratified into high-risk group and received all courses of the ALL-BFM 95 HR protocol with the exception of the induction phase.

b Unable to determine ‘day eight’ since prednisolone monotherapy was transiently terminated due to tumor lysis syndrome.

The peripheral blood count decreased rapidly to <1,000/μl following initiation of prednisolone. WBC, white blood cell count; PB, peripheral blood; SR, standard-risk; IR, intermediate-risk; HR, high-risk; RFS, relapse-free survival; IQR, interquartile range; ND, no data.

**Table V:** Results of univariate analysis by log-rank test.

Variables	n	HR (95% CI)	P-value
Age, years			
≤11	11	1	
>11	8	1.26 (0.21–7.40)	0.800
Gender			
Female	8	1	
Male	11	1.07 (0.18–6.37)	0.940
Immunophenotype of leukemic blasts			
B-lineage	9	1	
T-lineage	10	1.37 (0.23–8.02)	0.730
Initial leukocyte count, ×10^9^/l			
≤52.9	10	1	
>52.9	9	5.45 (0.85–35.0)	0.083
Response to prednisolone monotherapy (day 8 PB blast of <1,000/μl)			
Good	16	1	
Poor	3	1.38 (0.14–13.8)	0.770
Mean minimum leukocyte count/μl			
≤433	11	1	
>433	8	6.61 (1.04–42.1)	0.047

Median age, 11 years; median initial leukocyte count, 52.9×10^9^/l; median of the mean minimum leukocyte count, 433/μl. HR, hazard ratio; CI, confidence interval; PB, peripheral blood.
